# HPV genotype distribution and anomalous association of HPV33 to cervical neoplastic lesions in San Luis Potosí, Mexico

**DOI:** 10.1186/s13027-016-0063-z

**Published:** 2016-03-30

**Authors:** Raúl DelaRosa-Martínez, Mireya Sánchez-Garza, Rubén López-Revilla

**Affiliations:** División de Biología Molecular, Instituto Potosino de Investigación Científica y Tecnológica, Camino a la Presa San José 2055, 78216 San Luis Potosí, S.L.P., México

**Keywords:** Cervical cancer, Human papillomavirus, Atypical squamous cells of undetermined significance, Low-grade squamous intraepithelial lesions, High-grade squamous intraepithelial lesions, Invasive cervical carcinoma, HPV33 outbreak

## Abstract

**Background:**

The association of human papillomavirus (HPV) types to neoplastic lesions increase as a function of their oncogenicity and the duration of the infection since lesion severity progresses from low-grade to high-grade and cancer. In an outbreak, the prevalence of the HPV type involved would increase and the proportion of the associated low-grade lesions would predominate over severe lesions. In this study, the prevalence of HPV types and their association to neoplastic lesions was determined in women subjected to colposcopy in San Luis Potosí, Mexico.

**Methods:**

DNA from high-risk (HR) and low-risk (LR) HPV types was identified by E6 nested multiplex PCR in cervical scrapes from 700 women with normal cytology, atypical squamous cells of undetermined significance (ASCUS), low-grade squamous intraepithelial lesions (LSIL), high-grade squamous intraepithelial lesions (HSIL) or invasive cervical cancer (CC).

**Results:**

Overall HPV-DNA prevalence was 67.7 %, that of HR-HPV was 63.1 %, and that of LR-HPV was 21.3 %. The highest prevalence (78.2 %) occurred in the 15–24 year group, whereas that of single infections was 52 % and that of multiple infections (i.e., by 2–6 HPV types) was 48 %. The most prevalent HR types were HPV33 (33.1 %), HPV16 (16.6 %), HPV18 and HPV51 (6.7 % each). HR-HPV prevalence was 29.6 % in normal cytology, 26.7 % in ASCUS, 63.3 % in LSIL, 68.2 % in HSIL, and 90.5 % in CC. Three prevalence trends for HR-HPV types were found in neoplastic lesions of increasing severity: *increasing* (LSIL < HSIL < CC) for HPV16, HPV39, HPV18, HPV58, HPV31 and HPV35; *asymptotic* (LSIL < HSIL ≈ CC) for HPV51 and HPV68; *U-shaped* (LSIL < HSIL > CC) for HPV33.

**Conclusions:**

Two-thirds of the women subjected to colposcopy from 2007 to 2010 in San Luis Potosí have HPV infections which predominate in the 15–24 years group. Around half of the infections are by one viral type and the rest by 2–6 types. HPV33 is the most prevalent type, followed by HPV16. Overall HR-HPV prevalence increases with the severity of neoplastic lesions. HPV33 prevalence is highest in LSIL and its *U-shaped* trend with progressing neoplastic lesions differs from the *growing*/*asymptotic* trends of other HR-HPV types. An ongoing or recent HPV33 outbreak is consistent with its high prevalence and anomalous association to LSIL.

**Electronic supplementary material:**

The online version of this article (doi:10.1186/s13027-016-0063-z) contains supplementary material, which is available to authorized users.

## Background

Cervical cancer (CC) is the second cause of death by cancer among Mexican women [1]. In Mexico, 4031 deaths due to CC were reported in 2008 and the mortality rate was 9.7 per 100,000 women [2], around three times higher than that in developed countries [3]. In the Mexican state of San Luis Potosí, where this study was conducted, the CC mortality rate in 2008 was 9.3 per 100,000 women, similar to the national mortality rate [2].

Persistent infection of the cervical epithelium by anogenital human papillomavirus (HPV) types induces neoplastic lesions that may progress to invasive cancer [4, 5]. Among the 40 anogenital HPV types, the low-risk (LR-HPV) ones are associated to benign tumors and the high-risk (HR-HPV) ones to CC [6]. Two high-risk types, HPV16 and HPV18, cause around 70 % of CC cases worldwide [7] whereas two low-risk types, HPV6 and HPV11, cause 95 % of the anogenital wart cases [8].

The prevalence of HPV types infecting the cervix serves to predict the efficacy of the vaccines that prevent both HPV infections and cervical neoplastic lesions [9]. Although the prevalence of specific HPV types varies with the geographic region and the severity of neoplastic lesions [9–11], international meta-analyses show that HPV16 and HPV18 are the most prevalent high-risk types associated to cervical neoplastic lesions and CC [12, 13].

A previous study of our group on cervical infections by HR-HPV types in women from the capital city of San Luis Potosí demonstrated that HPV16 had the highest prevalence, 59 times that of HPV33 [14]. The present study identified 13 HR-HPV and six LR-HPV types in cervical scrapes of women from the six state sanitary jurisdictions that were subjected to colposcopy between 2007 and 2010. HPV infections were found in two-thirds of them, with three times more HR-HPV than LR-HPV types. The predominant type was HPV33, whose prevalence doubled that of HPV16, the second most prevalent type. The association of all HR-HPV types to neoplastic lesions increased as a function of their severity, except for HPV33, whose prevalence was highest in LSIL. An ongoing HPV33 outbreak is consistent with its high prevalence and anomalous association to neoplastic lesions.

## Methods

### Study design

This cross-sectional study of women attending the five colposcopy clinics of the state of San Luis Potosí was approved by the Ethics and Research Committee of the State Health Services. Informed consent from each participant included her authorization for colposcopy, cervical scraping and biopsy sampling, HPV DNA detection and genotyping and the use of clinical and sociodemographic data. Personal data of the participants are strictly confidential. The official Mexican guidelines for cervical cancer prevention, diagnosis, treatment, control and surveillance are mandatory at all Mexican hospitals and authorize health personnel to take samples both for cervical cytology and HPV infection tests to any sexually active woman less than 18 years old who requests them. Women are referred to colposcopy if a cervical anomalous result is diagnosed through gynecological examination or cytology; HPV tests are offered to women subjected to colposcopy and may be performed through hybrid capture or PCR.

### Setting

Recruitment was based on the availability of sociodemographic and clinical data, and of the cervical cytological and histopathological diagnoses of the women attending the clinics located at the Health Center of the capital city, the general hospitals of Matehuala, Rioverde, and Ciudad Valles, and the Tamazunchale Community Hospital, between June 2007 and August 2010.

### Participants

Eight hundred and thirty-seven 15–85 years-old women were eligible for the study. Those with incomplete or missing clinical records, inadequate conditions for cervical scraping (menstruation, pregnancy), or refusing to participate were excluded. Those with complete records, cytologic diagnosis before colposcopy, histopathologic diagnosis after colposcopy and informed consent were included.

### Variables

The variables analyzed were age at the time of the cervical scraping, prevalence of the HPV types identified and the cytologic and histopathologic diagnoses formulated with the 2001 Bethesda System [15]: normal, atypical squamous cells of undetermined significance (ASCUS), low-grade squamous intraepithelial lesions (LSIL), high-grade squamous intraepithelial lesions (HSIL) and cervical cancer (CC). “Normal” diagnosis corresponded to women seen for the first time or followed up. Histopathologic diagnoses of cervical intraepithelial neoplasia (CIN), formulated with Richart [16] nomenclature were reclassified as follows: CIN1 as LSIL, CIN2/CIN3 as HSIL [15]. Prevalence of each DNA HPV type was correlated with the severity of cervical neoplastic lesions.

### Sample size

The minimum size of a significant random sample (*n* = 682 cases), calculated with the OpenEpi 2.3.1 software [17], was based on the 1080 CIN and CC cases recorded during 2006 in the state of San Luis Potosí [2] assuming 50 % HPV prevalence, a 99 % confidence interval and a 3 % error.

### Cervical scrapes

From every woman an expert gynecologist took a cervical scrape for cytology, and less than two months later another one for the HPV test that was immediately placed in a tube containing 2 mL of fixative (1 mL of phosphate buffered saline supplemented with ethylenediamine tetraacetate and 1 mL of 96 % ethanol). Fixed scrapes were short term stored, transported at room temperature and maintained in the laboratory at 4 °C until DNA was extracted [14].

### DNA extraction and quantification

Each fixed sample was centrifuged at 13,000 rpm (16,250 × *g*) for 5 min in a Mikro 20 centrifuge (Hettich, Cologne, Germany). The supernatant was discarded and to the pellet were added 500 μL of TES (10 mM Tris-HCl, 2 mM EDTA, 0.4 M NaCl, pH 8.0), 50 μL of 10 % SDS and 20 μL of proteinase K (20 mg/mL). The mixtures were incubated 3 h at 56 °C in a thermoblock to digest proteins. After this incubation, 151 μL of 5 M NaCl were added and each mixture was centrifuged 15 min at 16,250 × *g*. The supernatant was carefully aspirated, transferred to a new tube to which 577 μL of isopropanol at −20 °C were added, and allowed to stand 10 min at 4 °C to precipitate the DNA. The tube was centrifuged at 16,250 × *g* for 10 min and the supernatant discarded by decantation [14].

The DNA pellet was washed twice with 1 mL of 70 % ethanol at room temperature and centrifuged 1 min at 10,000 rpm (9615 × *g*). The supernatant was carefully discarded by decantation and the pellet allowed to dry at room temperature for 15 min with the tube inverted on a paper towel. Pelleted DNA was dissolved with 50 or 100 μL of TE (10 mM Tris-HCl, 1 mM EDTA, pH 8.0) and 2 μL were applied to the Epoch Micro-Volume Spectrophotometer System (BioTek Instruments, Winooski, VT, USA) to determine optical densities at 260 and 280 nm.

### HPV detection and genotyping

The procedure was performed in 25 μL mixtures using the nested multiplex PCR E6 gene amplification method of Sotlar et al. [18], which identifies 13 HR-HPV types and six LR-HPV types but does not differentiate HPV6 from HPV11. Genotyping is based on the size of the amplicons of the identifiable HPV types. Initial PCR mixtures contained the GPE6/E7 consensus oligonucleotides purchased from Integrated DNA Technologies (Coralville, IA, USA) which amplify a ~630 bp product from E6 viral genes. Nested PCR was carried out in four separate mixtures, each containing 1/25 volume of the initial PCR mixture and cocktails of type-specific nested oligonucleotide pairs, also purchased from Integrated DNA Technologies. To avoid contamination, aliquots of each reagent were kept and managed separately and each stage of the HPV test —DNA extraction, PCR, and electrophoresis— was performed by operators using new gloves in separate laboratory areas that were decontaminated each time after a group of samples had been processed.

The initial mixtures contained PCR buffer (50 mM KCl, 20 mM Tris-HCl, pH 8.3), 200 μM dNTPs (each of the four), 1.5 mM MgCl_2_, 0.3 μM GPE6/E7 consensus oligonucleotides (each) and 1 unit of *Taq* DNA polymerase (Invitrogen, Carlsbad, CA, USA). Nested PCR mixtures contained PCR buffer, 152 μM dNTPs, 2 mM MgCl_2_, 0.3 μM HPV-type-specific oligonucleotides (each) and 1 unit of *Taq* DNA polymerase (Invitrogen, Carlsbad, CA, USA). PCR mixtures for β-globin contained 0.3 μM PC04/GH20 oligonucleotides and the same final concentrations of PCR buffer, dNTPs, and Taq DNA polymerase as in nested PCR. Amplification of the 260 bp segment of the human β-globin gene served to assess the quality of DNA in the HPV-negative cases [19].

The four nested PCR mixtures contained a total of 18 oligonucleotide pairs specific for 13 HR-HPV types (HPV16, HPV18, HPV31, HPV33, HPV35, HPV39, HPV45, HPV51, HPV52, HPV56, HPV58, HPV59, and HPV68) and six BR-HPV types (HPV6/11, HPV42, HPV43, HPV44 and HPV66). Mixtures without DNA were used as negative controls, whereas mixtures with DNA of the SiHa cell line (transformed by HPV16), DNA of the HeLa cell line (transformed by HPV18) or the pHPV33 plasmid containing the complete HPV33 genome were used as positive controls.

Incubations were performed in a 2720 thermocycler (Applied Biosystems, Foster City, CA, USA) under the following conditions. Starting PCR mixtures: initial denaturation at 94 °C for 4 min; 40 cycles at 94 °C for 1 min, 40 °C for 1 min and 72 °C for 2 min and a final extension at 72 °C for 10 min. Nested and β-globin PCR mixtures: initial denaturation at 94 °C for 4 min; 35 cycles at 94 °C for 30 s, 56 °C for 30 s, 72 °C for 45 s and a final extension at 72 °C for 4 min.

Five-μL of nested or β-globin PCR mixtures were applied to SB-2 % agarose [20] 5-mm thick gels run at 120 V for 35 min, stained with ethidium bromide and visualized under ultraviolet light in a ChemiDoc photodocumentation system (Bio-Rad, Hercules, CA, USA).

### Statistical analysis

Was performed with the Epi Info 7.1.1.14 software [21]. Age was expressed as the mean and standard deviation (SD) and as a categorical variable (age groups: 15–24, 25–34, 35–44, 45–54 and ≥55 years). The grades of neoplastic lesions were treated as absolute measures or proportions. HPV DNA prevalence (overall, of HR-HPV types, of LR-HPV types and of each of the HPV types identified in single and multiple infections) were calculated with 95 % confidence intervals (CI). Prevalences of single and multiple HPV types were calculated independently. HPV DNA categories: general, HR-HPV types, LR-HPV, single and multiple infections, were stratified by age groups (15–24, 25–34, 35–44, 45–54 and ≥55 years) and the prevalence in each group calculated with 95 % CI. The same HPV DNA categories were also stratified by the severity of neoplastic lesions, and the prevalence of groups with normal, ASCUS, LSIL, HSIL and CC diagnoses were calculated with 95 % CI. Single and multiple infections were stratified by the severity of neoplastic lesions and the corresponding prevalence calculated for each lesion group.

## Results

### Sociodemographic and clinical features of the participants

**Fig. 1:**
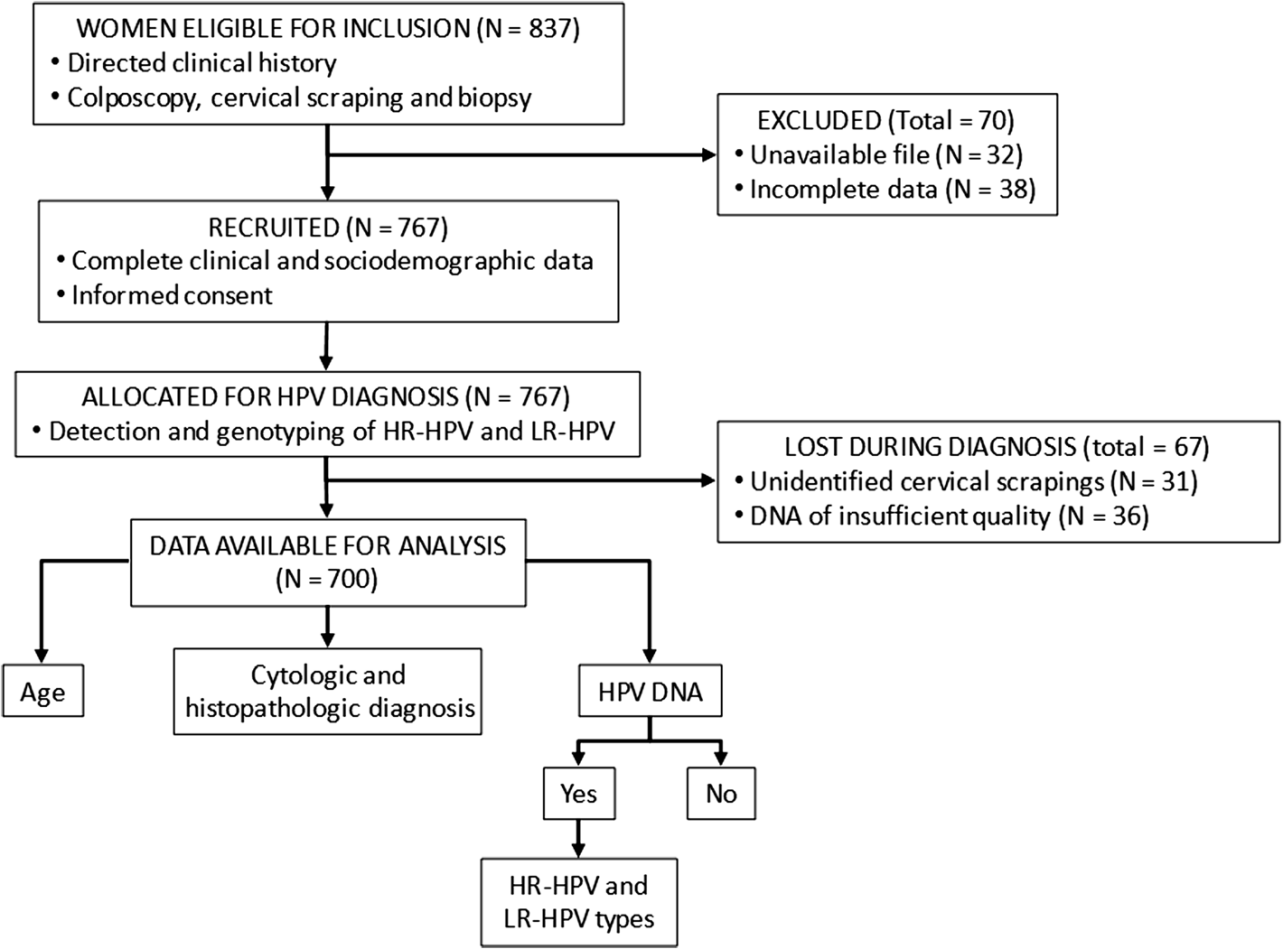
Flow diagram of the study

The 837 women subjected to colposcopy were assessed for recruitment. Seventy were excluded for having incomplete or no medical records. Among the 767 women recruited and allocated to HPV diagnosis, 67 were excluded for having unidentified cervical scrapes (*n* = 31) or DNA of insufficient quality (*n* = 36) (Fig. 1).

The study included the remaining 700 women 15 to 82 years old (mean age ± SD = 37.4 ± 12.0 years) with the following diagnoses: normal cytology (*n* = 27, 3.9 %), ASCUS (*n* = 15, 2.1 %), LSIL (*n* = 489, 69.9 %), HSIL (*n* = 148, 21.1 %) and CC (*n* = 21, 3.0 %) (Additional file 1: Table S1).

### Overall, HR- and LR-HPV DNA prevalence

Overall HPV DNA prevalence was 67.6 %, with 52.0 % of the cases having a single viral type (“single infection”) and 48.0 % having multiple viral types (“multiple infection”). HR-HPV DNA prevalence was 63.1 %, and LR-HPV DNA prevalence was 21.3 % (Additional file 1: Table S1).

**Fig. 2:**
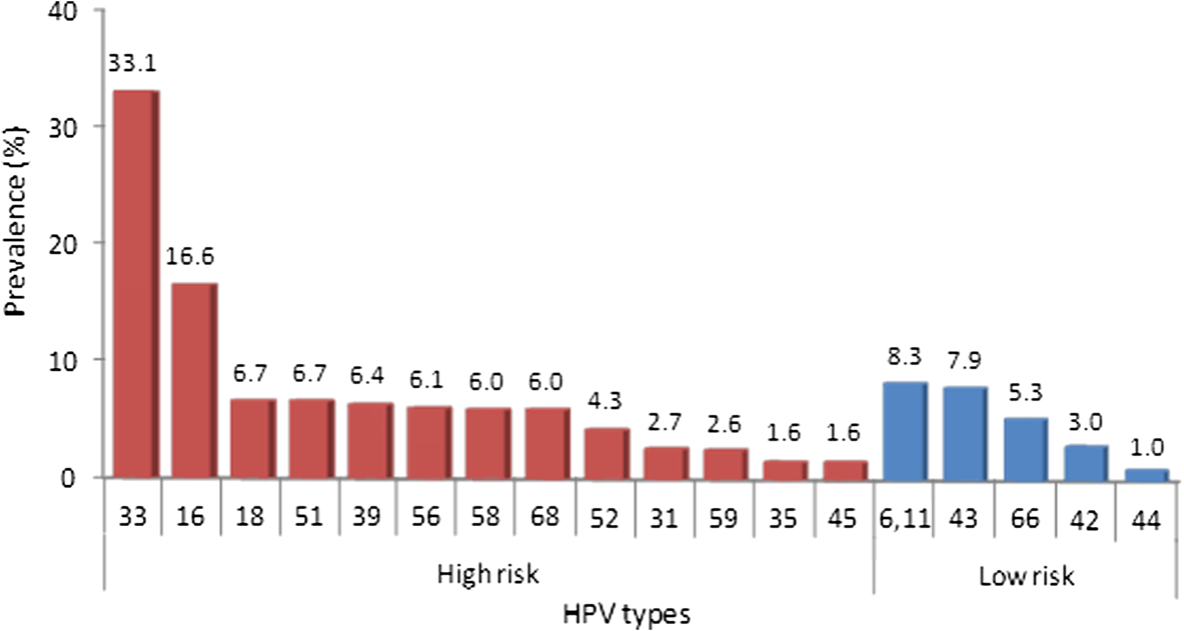
Prevalence of HR-HPV and LR-HPV types. The prevalence values of HR-HPV types (red bars) and LR-HPV types (*blue bars*) are presented in descending order

The most prevalent HR-HPV types were, in descending order, HPV33 (33.1 %), HPV16 (16.6 %), HPV18 (6.7 %) and HPV51 (6.7 %). The most prevalent LR-HPV types were HPV6/11 (8.3 %), HPV43 (7.9 %) and HPV66 (5.3 %) (Fig. 2, Additional file 1: Table S1).

**Fig. 3:**
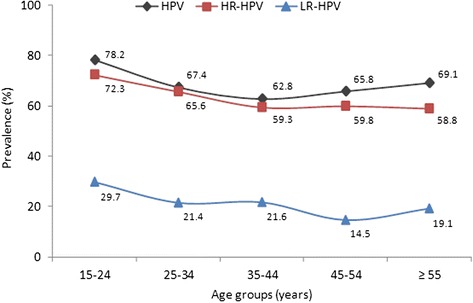
HPV DNA prevalences by age groups: overall, HR-HPV types and LR-HPV types. The overall HPV prevalence trend has a slightly pronounced U shape with its highest value in the 15–24 years group (78.2 %), followed by that of the ≥55 years group (69.1 %)

The highest overall HPV DNA prevalence (78.2 %) occurred in the 15–24 years group; decreased to 67.4 and 62.8 % in the 25–34 and 35–44 years groups, respectively; increased slightly to 65.8 %) in the 45–54 years group and to 69.1 % in the ≥55 years group (Fig. 3, Additional file 1: Table S2).

The highest HR-HPV DNA prevalence (72.3 %) occurred in the 15–24 years group, decreased to 65.6 % in the 25–34 years group and was slightly lower and similar in the groups of 35–44, 45–54 and ≥55 years groups (59.3, 59.8 and 58.8 %, respectively) (Fig. 3, Additional file 1: Table S2).

The highest LR-HPV DNA prevalence (29.7 %) corresponded to the 15–24 years group, decreased to 21.4 % in the 25–34 years group, was maintained in the 35–44 years group (21.6 %), reached its minimum value in the 45–54 years group (14.5 %) and increased slightly to 19.1 % in the ≥55 years group (Fig. 3, Additional file 1: Table S2).

The most prevalent HR-HPV type in all age groups was HPV33, followed by HPV16. The third most prevalent type varied with age: HPV56 in the 15–24 years group, HPV51 in 25–34 years group HPV52 in 35–44 years group, HPV18 in 45–54 years group, HPV68 in the ≥55 years group (Additional file 1: Table S2).

HPV33 prevalence was similar in the 15–24 years (35.6 %) and 25–34 years (35.3 %) groups, decreased in the 35–44 years group (28.6 %), was highest in the 45–54 years group (38.5 %), and decreased in the ≥ 55 years group (26.5 %). In contrast to HPV33 prevalence, the highest HPV16 prevalence (20.8 %) occurred in the 15–24 years group, was slightly lower but similar in the 25–34 and 35–44 years groups (16.8 % and 18.1 %, respectively), decreased to 8.5 % in the 45–55 years group, and increased in the ≥55 years group (20.6 %) to a value similar to that of the 15–24 years group (Additional file 1: Table S2).

### Overall, HR- and LR-HPV DNA prevalence in neoplastic lesions

**Fig. 4:**
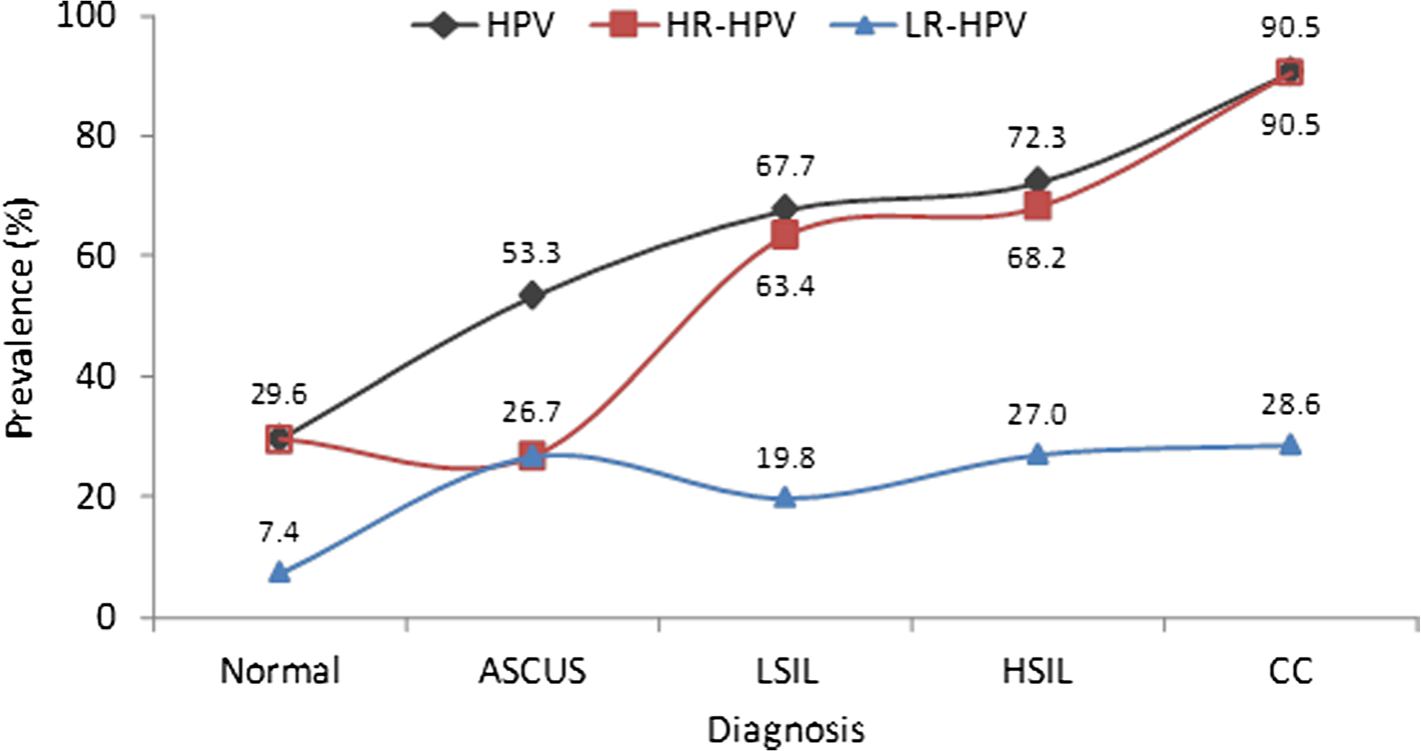
HPV DNA prevalences in neoplastic lesions: overall, HR-HPV types and LR-HPV types. Overall and HR-HPV prevalences increased with the severity of neoplastic lesions

Overall HPV DNA prevalence was 29.6 % in cases with normal cytology, 53.3 % in ASCUS, 67.7 % in LSIL, 72.3 % in HSIL and 90.5 % in CC (Fig. 4, Additional file 1: Table S3).

HR-HPV DNA prevalence was close to the overall HPV DNA prevalence found in lesions of increasing severity, except for ASCUS (29.6 % in cases with normal cytology, 26.7 % in ASCUS), 63.4 % in LSIL, 68.2 % in HSIL and 90.5 % in CC. In contrast, LR-HPV DNA prevalence was only 7.4 % in cases with normal cytology and increased to 26.7 and 28.6 % in ASCUS and CC (Fig. 4, Additional file 1: Table S3).

**Fig. 5:**
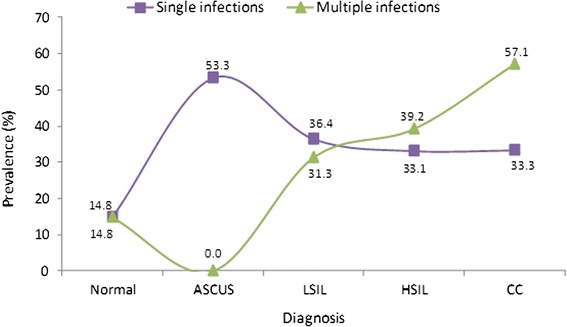
Prevalence of single and multiple HPV infections in neoplastic lesions. The prevalence of single infections was highest in ASCUS, decreased in LSIL and had similar values in HSIL and CC. The prevalence of multiple infections was null in ASCUS and increased gradually from LSIL to HSIL and CC

Prevalence of a single HPV DNA type was highest in ASCUS (53.3 %), clearly lower in cases with normal cytology (14.8 %), and intermediate and similar in LSIL, HSIL and CC (36.4, 33.1 and 33.3 %, respectively) (Fig. 5, Additional file 1: Table S3). Multiple HPV DNA types were not found in ASCUS, whereas their prevalence was 14.8 % in normal cytology cases (Fig. 5, Additional file 1: Table S3). In contrast with the similar prevalence of single HPV types in neoplastic lesions of different grades, the prevalence of multiple types increased as the severity of neoplastic lesions increased from 31.3 % in LSIL, 39.2 % in HSIL and 57.1 % in CC (Fig. 5, Additional file 1: Table S3).

### HPV types by age group and severity of neoplastic lesions

**Fig. 6:**
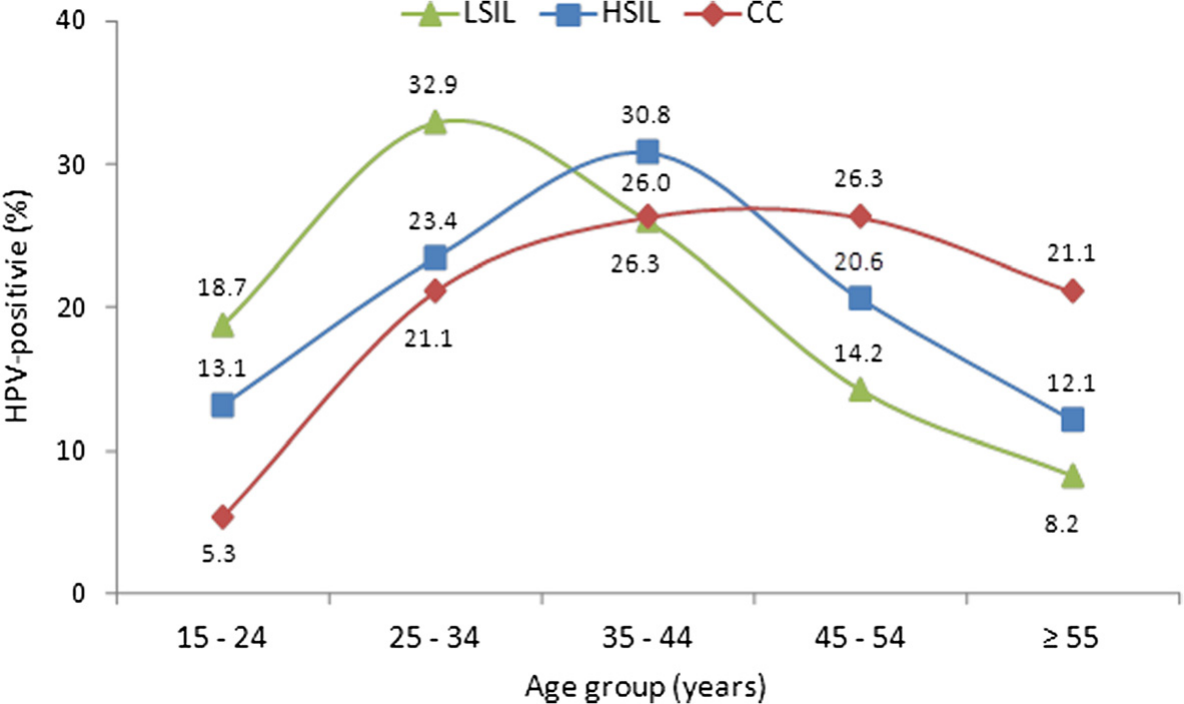
HPV-positivity in neoplastic lesions by age groups. Prevalences of HPV-positive cases were highest for LSIL in the 25–34 years group, for HSIL at the 35–44 years group and for CC in the 35–54 years group

The prevalence of HPV-positive cases associated to LSIL, HSIL and CC increased from the 15–24 years to the 25–34 years groups (Fig. 6). In LSIL it increased before, reaching their maximum value in the 25–34 years group and then decreasing gradually in the older age groups (Fig. 6). HPV-positivity in HSIL peaked in the 35–44 years group and declined in older age groups, whereas in CC it peaked in a plateau spanning the 35–44 and 45–54 years decades and then decreased slightly in the ≥55 years group (Fig. 6).

The most prevalent HR-HPV types in cases with normal cytology were HPV33 (18.5 %), HPV16 and HPV51 (11.1 %); in ASCUS the only types detected were HPV33 (13.3 %), and HPV39 and HPV51 (6.7 % each) (Additional file 1: Table S3).

In LSIL the most prevalent types were HPV33 (39.5 %) and HPV16 (14.9 %). In HSIL the most prevalent type was HPV16 (20.3 %), followed by HPV33 (16.3 %) and HPV39 (11.5 %) (Additional file 1: Table S3). The same types predominated in CC: HPV16 (47.6 %), HPV33 (38.1 %) and HPV39 (19.0 %). HPV18 ranked fourth in prevalence in LSIL, HSIL and CC (Additional file 1: Table S3).

**Fig. 7:**
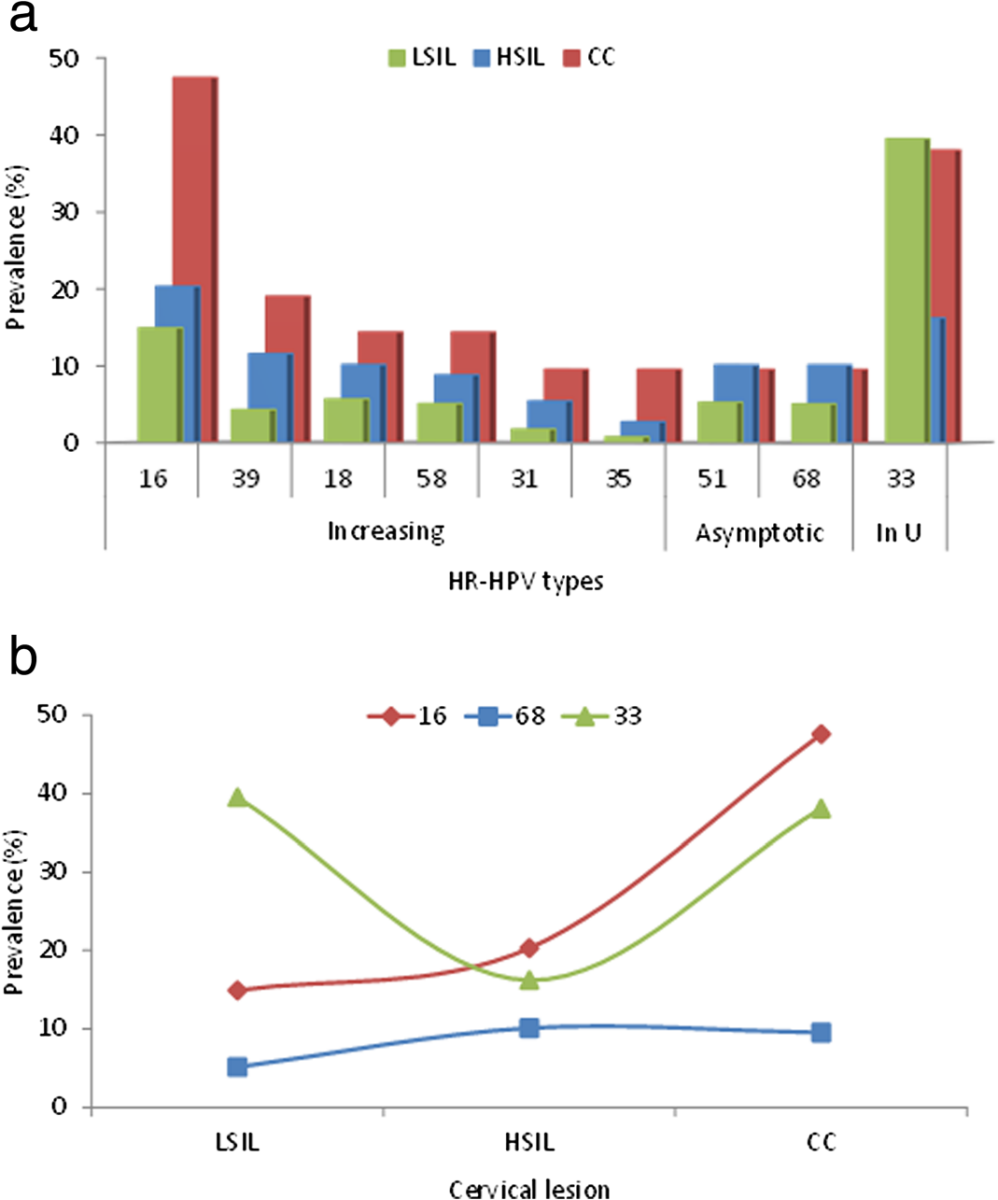
HR-HPV types with increasing, asymptotic and U-shaped prevalence trends for neoplastic lesions of increasing severity. **a** HR-HPV types with the three groups of trends (increasing, asymptotic, U-shaped) are placed in the x-axis in descending order of prevalence of the CC cases. The height of the bars is proportional to the prevalence of each viral type in lesions of increasing severity: LSIL (*green*), HSIL (*blue*) and CC (*red*). **b** Examples of prevalence trends of three HR-HPV types as a function of the severity of neoplastic lesions: *increasing* for HPV16, *asymptotic* for HPV68 and *U-shaped* for HPV33

Three prevalence trends as a function of neoplastic progression were identified among HR-HPV types (Fig. 7a): 1) *increasing* (minimum in LSIL, intermediate in HSIL, maximum in CC) for HPV16, HPV39, HPV18, HPV58, HPV31, and HPV35; 2) *asymptotic* (minimum in LSIL, maximum with similar values in HSIL and CC) for HPV51 and HPV68; and 3) *U-shaped* (maximum in LSIL and CC, minimum in HSIL) only for HPV33. Such prevalence trends for HPV16, HPV68 and HPV33 are illustrated in Fig. 7b.

The most prevalent LR-HPV types in LSIL were HPV43 (7.8 %), HPV6/11 (7.2 %) and HPV66 (4.9 %); in HSIL they were HPV6/11 (13.5 %) and HPV43 = HPV66 (7.4 %); and in CC they were HPV6/11 (14.3 %) and HPV43 = HPV44 (9.5 %) (Additional file 1: Table S3).

## Discussion

This is the first study on the prevalence of cervical infections by anogenital HR- and LR-HPV types and their association with the age and severity of neoplastic lesions in women subjected to colposcopy in the state of San Luis Potosí, Mexico. HPV33 appeared to be causing an outbreak at the time of the study since it was the most prevalent and the only HR-HPV type with the maximum association to low-grade neoplastic lesions.

Two-thirds (67.6 %) of the women included in the study were infected by HPV, a prevalence five to six times higher than that reported for women in the general population of Mexico [22] and other countries [23].

Nearly half of the HPV-positive women (47.9 %) had multiple infections, with a prevalence value in the upper end of the range of international studies [24].

In the state of San Luis Potosí circulate all 19 HPV types identifiable by the nested multiplex PCR method [18] used in this study. The order of prevalence of the major HR-HPV types (HPV33 > HPV16 > HPV18 = HPV58) contrasts with that reported in international studies (HPV16 > HPV18 > HPV31) [10, 12]. It is especially striking that in this study HPV33 prevalence duplicates that of HPV16.

The most prevalent LR-HPV types were HPV6/11 > HPV43 > HPV66. Although the typing method used does not distinguish HPV6 from HPV11, international and Mexican studies suggest that HPV6 may be the most prevalent [25]. The order of prevalence found for HR-HPV types differs from those described in global and South and Central America studies [13] as well as in other Mexican regions [26, 27].

The overall HPV prevalence found by us is higher than that reported for women of the general population or with normal cytology, and the age-specific prevalence curve is U-shaped, as expected, although less pronounced than the curves found in international studies of the general population [28–30]. Both differences are presumably due to the fact that the women included in this study were not from the general population but had been previously diagnosed with cervical neoplastic lesions. The highest prevalence values at both ends of the U curve correspond to the 15-24 years (78.0 %) and ≥55 years (69.0 %) groups, and are similar to those previously found in Mexico [22] and Latin America [12, 28, 30].

The overall HPV infection determined correlates with the severity of neoplastic lesions as has been observed in international studies [13, 31] and in Mexico [26]. The HPV prevalence found by us in cases without lesions (29.6 %) is also within the range found in international studies [23] and in Mexico [22, 26]. Furthermore, the prevalence of HR-HPV types found by us in CC (90.5 %) is similar to that of global studies [31] and consistent with the concept that persistent HR-HPV infection is a necessary cause for CC [5, 10].

The discordance between the diagnosis of neoplastic lesion and HPV infection probably derives both from the cytological overdiagnosis in cases of apparent lesions without infection (32.4 %) and the lower sensitivity of cytology and colposcopy in cases of infection without apparent lesion (29.6 %). These results reinforce the notion that HPV DNA detection is preferable to cytology as the primary screening test for CC [32, 33].

In this study HPV33 was the most prevalent type in LSIL, whereas HPV16 is the most prevalent type in several multicentric international studies [13]. Moreover, the order of prevalence found by us in LSIL for HR-HPV types (HPV33 > HPV16 > HPV56) differs from that found by Clifford et al. [13] in HSIL and CC (HPV16 > HPV33 > HPV39), and indicates that HPV16 is also the most oncogenic type in the state of San Luis Potosí.

A previous study of cervical samples taken between 2004 and 2005 from women of the city of San Luis Potosí with similar sociodemographic and clinical characteristics to those of the present study found that HPV16, HPV31 and HPV18 were the predominant high-risk types [14]. HPV33 prevalence in the capital city (1.1 %) compared with that of the present study (23.6 %) corresponds to a 21.4-fold increase and is consistent with an outbreak that may have originated from several municipalities located in the middle region of the state whose average HPV33 prevalence in the present study is 56.2 % (data not shown). Since the severity of cervical neoplastic lesions progresses as the duration of HPV infections increases, in a steady-state epidemiologic situation (i.e., with no outbreaks by any viral type) the prevalence trends of the HR-HPV types as a function of the severity of neoplastic lesions would be *increasing* (i.e., LSIL > HSIL > CC) for the more oncogenic types and *asymptotic* for the less oncogenic ones (i.e., LSIL > HSIL = CC). In the present study both trends were found for all the HR-HPV types identified except for HPV33, whose highest prevalence was in LSIL, the most recent lesions. Besides its predominance, HPV33 was also the only viral type whose prevalence as a function of the severity of neoplastic lesions was not increasing or asymptotic but U-shaped (i.e., higher in LSIL and CC and minimum in HSIL). Both of these anomalies suggest that an HPV33 outbreak was taking place during the sampling for the present study.

A recent study performed by Chen et al. [34] in 30 countries discovered new HPV33 variants that were classified in four phylogenetic groups. We are currently sequencing the ORF E6 from HPV33 single infections in order to identify its circulating variants and to determine their geographic distribution and network connections.

Current prophylactic vaccines prevent both the infection and development of neoplastic lesions caused by the HPV types to which they are directed [35]. They also induce partial protection lasting six months to three years against a limited number of HR-HPV types phylogenetically related to HPV16 or HPV18 [36]: Cervarix® against HPV31, HPV33, HPV45 and HPV52, and Gardasil® against HPV31 [35]. The prevalence of the circulating HPV types found in this study suggests that both vaccines could have prevented 23 % of the infections, as well as 25 % of the HSIL and 62 % of the CC due to HPV16 and HPV18 among the women included in the study.

This work is being complemented by the identification of the HPV types present in paraffin blocks of CC specimens to estimate the prevalence and relative oncogenicity of the HPV types as well as which prophylactic multivalent vaccines would be most effective in this region.

## Conclusions

Two-thirds of the women referred to colposcopy in San Luis Potosí, Mexico, were infected by all 19 HPV types identifiable with the method used. Overall HPV prevalence was higher than that expected in the general population because the women included in the study had been shown to have abnormal cytology results. The unexpected order of prevalence of the major HR-HPV types was HPV33 > HPV16 > HPV18 = HPV58. HPV33 appeared to be causing an outbreak at the time of the study because it was the most prevalent and the only HR-HPV type whose association to LSIL predominated. The prevalence of the circulating HPV types and their association to neoplastic lesions suggest that the divalent and quadrivalent prophylactic anti-HPV vaccines could have prevented 23 % of the infections as well as 25 % of the HSIL and 62 % of the CC associated to HPV16 and HPV18.

## Additional file

Additional file 1: Table S1. Cervical neoplastic lesions of increasing severity by age groups (*N* = 700). **Table S2.** HPV DNA prevalence in age groups: overall, HR-HPV, LR-HPV, specific viral types, single infections and multiple infections (*N* = 700). **Table S3.** HPV DNA prevalence in neoplastic lesions: overall, HR-HPV, LR-HPV, specific viral types, single infections and multiple infections (*N* = 700). (DOCX 22 kb)

## Abbreviations

ASCUS: atypical squamous cells of undetermined significance
CC: cervical cancer
CIN: cervical intraepithelial neoplasia
CONACYT: National Council for Science and Technology (Mexico)
HPV: human papillomavirus
HR: high-risk
HSIL: high-grade squamous intraepithelial lesion
LR: low-risk
LSIL: low-grade squamous intraepithelial lesion
