# Supplementary material for: HPV genotype distribution and anomalous association of HPV33 to cervical neoplastic lesions in San Luis Potosí, Mexico
Source: Infect Agent Cancer. 2016 Mar 30;11:16. doi: 10.1186/s13027-016-0063-z (PMC4812629; doi:10.1186/s13027-016-0063-z)
Supplement: Additional file 1: Table S1. — Cervical neoplastic lesions of increasing severity by age groups (N = 700). Table S2. HPV DNA prevalence in age groups: overall, HR-HPV, LR-HPV, specific viral types, single infections and multiple infections (N = 700). Table S3. HPV DNA prevalence in neoplastic lesions: overall, HR-HPV, LR-HPV, specific viral types, single infections and multiple infections (N = 700). (DOCX 22 kb) [file 13027_2016_63_MOESM1_ESM.docx]

# Supplementary material (DelaRosa et al. IAAC MS)

Table S 1 Cervical neoplastic lesions of increasing severity by age groups (N = 700)

| **Group** | **Diagnosis** | | | | | |
| --- | --- | --- | --- | --- | --- | --- |
|  | **Normal** | **ASCUS** | **LSIL** | **HSIL** | **CC** | **Total** |
| Population, N (%) | 27 (3.9) | 15 (2.1) | 489 (69.9) | 148 (21.1) | 21 (3.0) | 700 (100.0) |
| Mean age (SD) | 40.8 (± 11.5) | 38.9 (± 11.0) | 35.9 (± 11.4) | 40.6 (± 12.8) | 45.9 (± 12.9) | 37.4 (± 12.0) |
| Distribution by age, N (%) |  |  |  |  |  |  |
| 15-24 | 1 (3.7) | 1 (6.7) | 81 (16.6) | 17 (11.5) | 1 (4.8) | 101 (14.4) |
| 25-34 | 8 (29.6) | 5 (33.3) | 162 (33.1) | 36 (24.3) | 4 (19.0) | 215 (30.7) |
| 35-44 | 8 (29.6) | 5 (33.3) | 140 (28.6) | 41 (27.7) | 5 (23.8) | 199 (28.4) |
| 45-54 | 6 (22.2) | 2 (13.3) | 71 (14.5) | 33 (22.3) | 5 (23.8) | 117 (16.7) |
| ≥ 55 | 4 (14.8) | 2 (13.3 | 35 (7.2) | 21 (14.2) | 6 (28.6) | 68 (9.7) |

## Table S 2 HPV DNA prevalence in age groups: overall, HR-HPV, LR-HPV, specific viral types, single infections and multiple infections (N = 700)

| **HPV infection categories and viral types** | **Overall prevalence (N = 700)** | | **HPV-positivity (N = 473)** | **Prevalence by age group (years)** | | | | | | | | | |
| --- | --- | --- | --- | --- | --- | --- | --- | --- | --- | --- | --- | --- | --- |
|  |  |  |  | **15-24 (N = 101)** | | **25-34 (N = 215)** | | **35-44 (N = 199)** | | **45-54 (N = 117)** | | **≥ 55 (N = 68)** | |
|  | **N** | **% (95% CI)** | **%** | **N** | **% (95% CI)** | **N** | **% (95% CI)** | **N** | **% (95% CI)** | **N** | **% (95% CI)** | **N** | **% (95% CI)** |
| Overall | 473 | 67.6 (63.9-71.0) | 100.0 | 79 | 78.2 (68.9-85.8) | 145 | 67.4 (60.7-73.7) | 125 | 62.8 (55.7-69.5) | 77 | 65.8 (56.5-74.3) | 47 | 69.1 (56.7-79.8) |
| HR-HPV types | 442 | 63.1 (59.4-66.7) | 93.4 | 73 | 72.3 (62.5-80.7) | 141 | 65.6 (58.8-71.9) | 118 | 59.3 (52.1-66.2) | 70 | 59.8 (50.4-68.8) | 40 | 58.8 (46.2-70.6) |
| HPV16 | 116 | 16.6 (13.9-19.6) | 24.5 | 21 | 20.8 (13.4-30.0) | 35 | 16.3 (11.6-21.9) | 36 | 18.1 (13.0-24.2) | 10 | 8.5 (4.2-15.2) | 14 | 20.6 (11.7-32.1) |
| HPV18 | 47 | 6.7 (5.0-8.9) | 9.9 | 7 | 6.9 (2.8-13.8) | 15 | 7.0 (4.0-11.2) | 11 | 5.5 (2.8-9.7) | 10 | 8.5 (4.2-15.2) | 4 | 5.9 (1.6-14.4) |
| HPV31 | 19 | 2.7 (1.7-4.3) | 4.0 | 1 | 1.0 (0.0-5.4) | 7 | 3.3 (1.3-6.6) | 5 | 2.5 (0.8-5.8) | 4 | 3.4 (0.9-8.5) | 2 | 2.9 (0.4-10.2) |
| HPV33 | 232 | 33.1 (29.7-36.8) | 49.0 | 36 | 35.6 (26.4-45.8) | 76 | 35.3 (29.0-42.1) | 57 | 28.6 (22.5-35.5) | 45 | 38.5 (29.6-47.9) | 18 | 26.5 (16.5-38.6) |
| HPV35 | 11 | 1.6 (0.8-2.9) | 2.3 | 1 | 1.0 (0.0-5.4) | 6 | 2.8 (1.0-6.0) | 2 | 1.0 (0.1-3.6) | 2 | 1.7 (0.2-6.0) | 0 | 0.0 (0.0-0.0) |
| HPV39 | 45 | 6.4 (4.8-8.6) | 9.5 | 4 | 4.0 (1.1-9.8) | 18 | 8.4 (5.0-12.9) | 10 | 5.0 (2.4-9.0) | 9 | 7.7 (3.6-14.1) | 4 | 5.9 (1.6-14.4) |
| HPV45 | 11 | 1.6 (0.8-2.9) | 2.3 | 2 | 2.0 (0.2-7.0) | 4 | 1.9 (0.5-4.7) | 3 | 1.5 (0.3-4.3) | 0 | 0.0 (0.0-0.0) | 2 | 2.9 (0.4-10.2) |
| HPV51 | 47 | 6.7 (5.0-8.9) | 9.9 | 8 | 7.9 (3.5-15.0) | 21 | 9.8 (6.1-14.5) | 8 | 4.0 (1.8-7.8) | 8 | 6.8 (3.0-13.0) | 2 | 2.9 (0.4-10.2) |
| HPV52 | 30 | 4.3 (3.0-6.1) | 6.3 | 5 | 5.0 (1.6-11.2) | 6 | 2.8 (1.0-6.0) | 12 | 6.0 (3.2-10.3) | 4 | 3.4 (0.9-8.5) | 3 | 4.4 (0.9-12.4) |
| HPV56 | 43 | 6.1 (4.5-8.3) | 9.1 | 12 | 11.9 (6.3-19.8) | 12 | 5.6 (2.9-9.5) | 9 | 4.5 (2.1-8.4) | 4 | 3.4 (0.9-8.5) | 6 | 8.8 (3.3-18.2) |
| HPV58 | 42 | 6.0 (4.4-8.1) | 8.9 | 6 | 5.9 (2.2-12.5) | 14 | 6.5 (3.6-10.7) | 11 | 5.5 (2.8-9.7) | 7 | 6.0 (2.4-11.9) | 4 | 5.9 (1.6-14.4) |
| HPV59 | 18 | 2.6 (1.6-4.1) | 3.8 | 2 | 2.0 (0.2-7.0) | 6 | 2.8 (1.0-6.0) | 7 | 3.5 (1.4-7.1) | 2 | 1.7 (0.2-6.0) | 1 | 1.5 (0.0-7.9) |
| HPV68 | 42 | 6.0 (4.4-8.1) | 8.9 | 7 | 6.9 (2.8-13.8) | 12 | 5.6 (2.9-9.5) | 11 | 5.5 (2.8-9.7) | 4 | 3.4 (0.9-8.5) | 8 | 11.8 (5.2-21.9) |
| LR-HPV types | 149 | 21.3 (18.3-24.5) | 31.5 | 30 | 29.7 (21-39.6) | 46 | 21.4 (16.1-27.5) | 43 | 21.6 (16.1-28) | 17 | 14.5 (8.7-22.2) | 13 | 19.1 (10.6-30.5) |
| HPV42 | 21 | 3.0 (1.9-4.6) | 4.4 | 5 | 5.0 (1.6-11.2) | 9 | 4.2 (1.9-7.8) | 5 | 2.5 (0.8-5.8) | 1 | 0.9 (0.0-4.7) | 1 | 1.5 (0.0-7.9) |
| HPV43 | 55 | 7.9 (6.0-10.2) | 11.6 | 15 | 14.9 (8.6-23.3) | 16 | 7.4 (4.3-11.8) | 12 | 6.0 (3.2-10.3) | 6 | 5.1 (1.9-10.8) | 6 | 8.8 (3.3-18.2) |
| HPV44 | 7 | 1.0 (0.4-2.1) | 1.5 | 1 | 1.0 (0.0-5.4) | 2 | 0.9 (0.1-3.3) | 2 | 1.0 (0.1-3.6) | 0 | 0.0 (0.0-0.0) | 2 | 2.9 (0.4-10.2) |
| HPV6/11 | 58 | 8.3 (6.4-10.6) | 12.3 | 10 | 9.9 (4.9-17.5) | 18 | 8.4 (5.0-12.9) | 17 | 8.5 (5.1-13.3) | 9 | 7.7 (3.6-14.1) | 4 | 5.9 (1.6-14.4) |
| HPV66 | 37 | 5.3 (3.8-7.3) | 7.8 | 8 | 7.9 (3.5-15.0) | 11 | 5.1 (2.6-9.0) | 12 | 6.0 (3.2-10.3) | 4 | 3.4 (0.9-8.5) | 2 | 2.9 (0.4-10.2) |
| Single infections | 246 | 35.1 (31.6-38.8) | 52.0 | 41 | 40.6 (30.9-50.8) | 65 | 30.2 (24.2-36.8) | 65 | 32.7 (26.2-39.7) | 50 | 42.7 (33.6-52.2) | 25 | 36.8 (25.4-49.3) |
| Multiple infections | 227 | 32.4 (29.0-36.1) | 48.0 | 38 | 37.6 (28.2-47.8) | 80 | 37.2 (30.7-44.0) | 60 | 30.2 (23.9-37.0) | 27 | 23.1 (15.8-31.8) | 22 | 32.4 (21.5-44.8) |

The prevalence of each viral type includes single and multiple infections. Confidence intervals of 95% are indicated in parentheses (95% CI).

## Table S 3 HPV DNA prevalence in neoplastic lesions: overall, HR-HPV, LR-HPV, specific viral types, single infections and multiple infections (N = 700)

| **HPV infection categories and viral types** | **Prevalence by severity of neoplastic lesions** | | | | | | | | | |
| --- | --- | --- | --- | --- | --- | --- | --- | --- | --- | --- |
|  | **Normal (N = 27)** | | **ASCUS (N = 15)** | | **LSIL (N = 489)** | | **HSIL (N = 148)** | | **CC (N = 21)** | |
|  | **N** | **% (95% CI)** | **N** | **% (95% CI)** | **N** | **% (95% CI)** | **N** | **% (95% CI)** | **N** | **% (95% CI)** |
| HPV (overall) | 8 | 29.6 (13.8-50.2) | 8 | 53.3( 26.6-78.7) | 331 | 67.7 (63.3- 71.8) | 107 | 72.3 (64.3-79.3) | 19 | 90.5 (69.6-98.8) |
| HR-HPV types | 8 | 29.6 (13.8-50.2) | 4 | 26.7 (7.8-55.1) | 310 | 63.4 (58.9- 67.6) | 101 | 68.2 (60.1-75.6) | 19 | 90.5 (69.6-98.8) |
| HPV16 | 3 | 11.1 (2.4-29.2) | 0 | 0.0 (0.0-0.0) | 73 | 14.9 (12.0-18.5) | 30 | 20.3 (14.1-27.7) | 10 | 47.6 (25.7-70.2) |
| HPV18 | 1 | 3.7 (0.1-19.0) | 0 | 0.0 (0.0-0.0) | 28 | 5.7 (3.9-8.3) | 15 | 10.1 (5.8-16.2) | 3 | 14.3 (3.0-36.3) |
| HPV31 | 0 | 0.0 (0.0-0.0) | 0 | 0.0 (0.0-0.0) | 9 | 1.8 (0.9-3.6) | 8 | 5.4 (2.4-10.4) | 2 | 9.5 (1.2-30.4) |
| HPV33 | 5 | 18.5 (6.3-38.1) | 2 | 13.3 (1.7-40.5) | 193 | 39.5 (35.1-44.0) | 24 | 16.2 (10.7-23.2) | 8 | 38.1 (18.1-61.6) |
| HPV35 | 1 | 3.7 (0.1-19.0) | 0 | 0.0 (0.0-0.0) | 4 | 0.8 (0.3-2.2) | 4 | 2.7 (0.7-6.8) | 2 | 9.5 (1.2-30.4) |
| HPV39 | 2 | 7.4 (0.9-24.3) | 1 | 6.7 (0.2-31.9) | 21 | 4.3 (2.7-6.6) | 17 | 11.5 (6.8-17.8) | 4 | 19.0 (5.4-41.9) |
| HPV45 | 0 | 0.0 (0.0-0.0) | 0 | 0.0 (0.0-0.0) | 7 | 1.4 (0.6-3.1) | 4 | 2.7 (0.7-6.8) | 0 | 0.0 (0.0-0.0) |
| HPV51 | 3 | 11.1 (2.4-29.2) | 1 | 6.7 (0.2-31.9) | 26 | 5.3 (3.6-7.8) | 15 | 10.1 (5.8-16.2) | 2 | 9.5 (1.2-30.4) |
| HPV52 | 0 | 0.0 (0.0-12.8) | 0 | 0.0 (0.0-0.0) | 20 | 4.1 (2.6-6.4) | 10 | 6.8 (3.3-12.1) | 0 | 0.0 (0.0-0.0) |
| HPV56 | 1 | 3.7 (0.1-19.0) | 0 | 0.0 (0.0-0.0) | 31 | 6.3 (4.4-9.0) | 11 | 7.4 (3.8-12.9) | 0 | 0.0 (0.0-0.0) |
| HPV58 | 1 | 3.7 (0.1-19.0) | 0 | 0.0 (0.0-0.0) | 25 | 5.1 (3.4-7.6) | 13 | 8.8 (4.8-14.6) | 3 | 14.3 (3.0-36.3) |
| HPV59 | 0 | 0.0 (0.0-0.0) | 0 | 0.0 (0.0-0.0) | 12 | 2.5 (1.3-4.4) | 6 | 4.1 (1.5-8.6) | 0 | 0.0 (0.0-00) |
| HPV68 | 0 | 0.0 (0.0-0.0) | 0 | 0.0 (0.0-0.0) | 25 | 5.1 (3.4-7.6) | 15 | 10.1 (5.8-16.2) | 2 | 9.5 (1.2-30.4) |
| LR-HPV types | 2 | 7.4 (0.9-24.3) | 4 | 26.7 (7.8-55.1) | 97 | 19.8 (16.4- 23.7) | 40 | 27.0 (20.1-34.9) | 6 | 28.6 (11.3-52.2) |
| HPV42 | 0 | 0.0 (0.0-0.0) | 0 | 0.0 (0.0-0.0) | 16 | 3.3 (1.9-5.4) | 5 | 3.4 (1.1-7.7) | 0 | 0.0 (0.0-0.0) |
| HPV43 | 1 | 3.7 (0.1-19.0) | 3 | 20.0 (4.3-48.1) | 38 | 7.8 (5.6-10.6) | 11 | 7.4 (3.8-12.9) | 2 | 9.5 (1.2-30.4) |
| HPV44 | 1 | 3.7 (0.1-19.0) | 0 | 0.0 (0.0-0.0) | 2 | 0.4 (0.1-1.6) | 2 | 1.4 (0.2-4.8) | 2 | 9.5 (1.2-30.4) |
| HPV6/11 | 0 | 0.0 (0.0-0.0) | 0 | 0.0 (0.0-0.0) | 35 | 7.2 (5.1-9.9) | 20 | 13.5 (8.5-20.1) | 3 | 14.3 (3.0-36.3) |
| HPV66 | 1 | 3.7 (0.1-19.0) | 1 | 6.7 (0.2-31.9) | 24 | 4.9 (3.2-7.3) | 11 | 7.4 (3.8-12.9) | 0 | 0.0 (0.0-0.0) |
| Single infections | 4 | 14.8 (4.2-33.7) | 8 | 53.3 (26.6-78.7) | 178 | 36.4 (32.2-40.9) | 49 | 33.1 (25.6-41.3) | 7 | 33.3 (14.6-57.0) |
| Multiple infections | 4 | 14.8 (4.2-33.7) | 0 | 0 (0.0-0.0) | 153 | 31.3 (27.2-35.6) | 58 | 39.2 (31.3-47.5) | 12 | 57.1 (34.0-78.2) |

The prevalence of each viral type includes single and multiple infections. Confidence intervals of 95% are indicated in parentheses (95% CI).
